# Hemodialysis catheter insertion: is increased PO_2_ a sign of arterial cannulation? A case report

**DOI:** 10.1186/1471-2369-15-127

**Published:** 2014-07-29

**Authors:** Julio C Chirinos, Javier A Neyra, Jiten Patel, Aylin R Rodan

**Affiliations:** 1 Department of Medicine, Division of Nephrology, University of Texas Southwestern Medical Center, 5323 Harry Hines Blvd, Dallas, Texas, USA

**Keywords:** Hemodialysis, Catheter, Central venous cannulation, Vein anomaly

## Abstract

**Background:**

Ultrasound-guided Central Venous Catheterization (CVC) for temporary vascular access, preferably using the right internal jugular vein, is widely accepted by nephrologists. However CVC is associated with numerous potential complications, including death. We describe the finding of a rare left-sided partial anomalous pulmonary vein connection during central venous catheterization for continuous renal replacement therapy (CRRT).

**Case presentation:**

Ultrasound-guided cannulation of a large bore temporary dual-lumen Quinton-Mahurkar catheter into the left internal jugular vein was performed for CRRT initiation in a 66 year old African-American with sepsis-related oliguric acute kidney injury. The post-procedure chest X-ray suggested inadvertent left carotid artery cannulation. Blood gases obtained from the catheter showed high partial pressure of oxygen (PO_2_) of 140 mmHg and low partial pressure of carbon dioxide (PCO_2_) of 22 mmHg, suggestive of arterial cannulation. However, the pressure-transduced wave forms appeared venous and Computed Tomography Angiography located the catheter in the left internal jugular vein, but demonstrated that the tip of the catheter was lying over a left pulmonary vein which was abnormally draining into the left brachiocephalic (innominate) vein rather than into the left atrium.

**Conclusion:**

Although several mechanical complications of dialysis catheters have been described, ours is one of the few cases of malposition into an anomalous pulmonary vein, and highlights a sequential approach to properly identify the catheter location in this uncommon clinical scenario.

## Background

Central venous catheterization (CVC) using a large bore catheter (>7 French) is widely used in renal patients for hemodynamic monitoring, rapid infusion of fluids and blood products, antibiotic administration, parenteral nutrition, and vascular access for hemodialysis and continuous renal replacement therapy (CRRT). Ultrasound (US)-guided CVC for temporary vascular access, preferably using the right internal jugular vein (IJV), is widely accepted by nephrologists [1]. Compared to the subclavian approach, right IJV is the preferred site because of easier catheterization, high rate of success when using only anatomical landmarks of the sternocleidomastoid muscle, and straighter path to the superior vena cava [2]. However CVC is associated with numerous potential complications, including death. Most mechanical complications occur early, during the puncture of the target vessel or catheter advancement, with subsequent development of hemorrhage, pseudoaneurysms, arteriovenous fistula, arterial dissection, neurological injury and severe or lethal airway obstruction [3,4]. Therefore, nephrologists and nephrology trainees should not only be trained in temporary vascular access placement, but also be informed about techniques to identify or differentiate successful venous punctures from arterial punctures, as well as how to prevent and manage procedure-related complications [1,2].

## Case presentation

**Figure 1:**
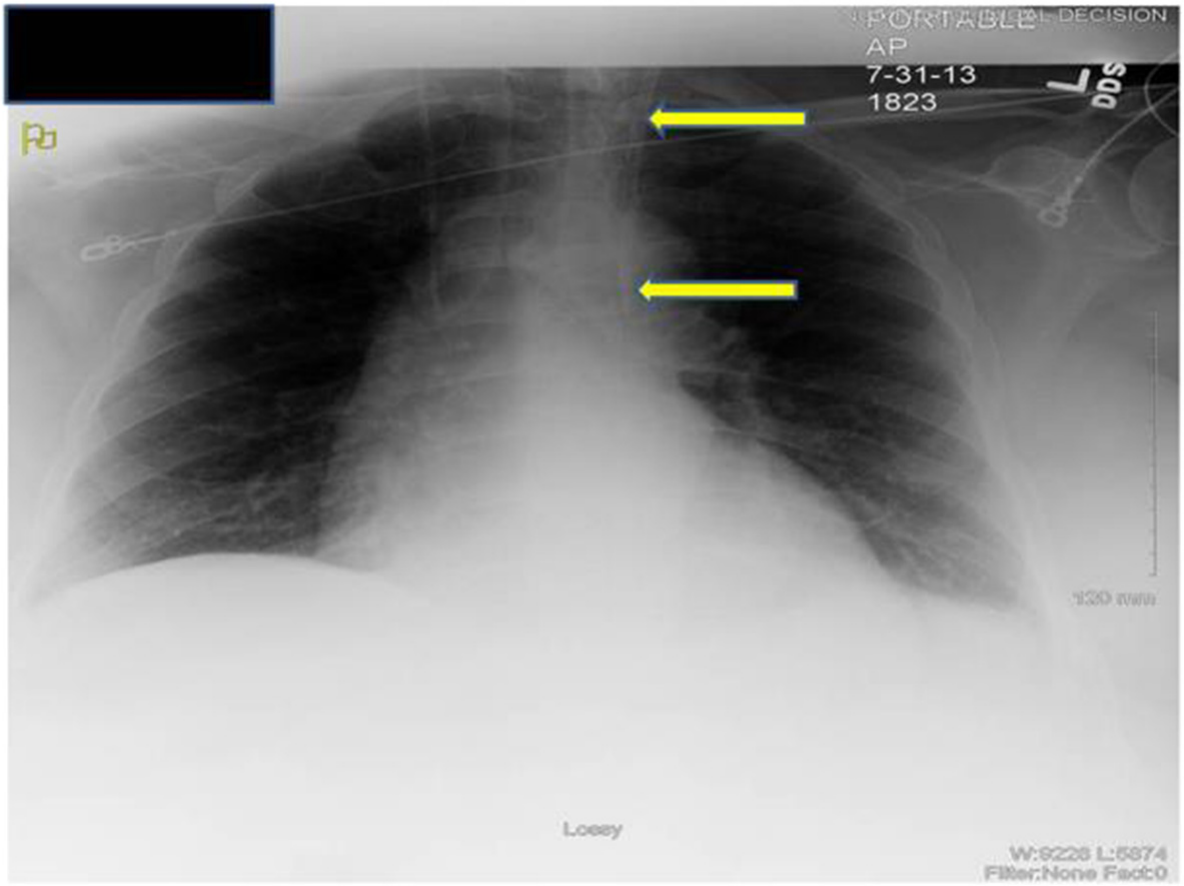
**Chest Radiography.** Chest-X ray showing the Quinton-Mahurkar catheter tip (yellow arrow) not crossing the midline to the right side.

**Figure 2:**
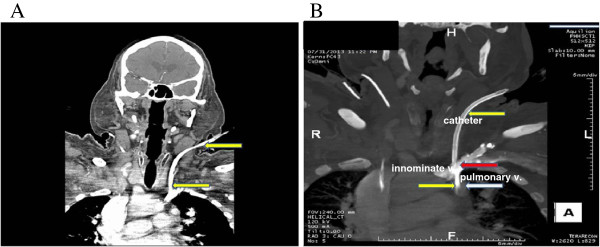
**Computed Tomography Angiography (CTA). (A)** CTA showing Quinton-Mahurkar catheter (yellow arrow) extending into the left IJV and subsequently descending to terminate in an anomalous left upper lobe pulmonary vein, just inferior to its anomalous insertion in the left brachiocephalic vein; **(B)** CTA showing Quinton-Mahurkar catheter (yellow arrow), left brachiocephalic (innominate) vein (red arrow) and anomalous pulmonary vein (white arrow) draining into the left brachiocephalic (innominate) vein.

A 66 year old man with underlying chronic kidney disease, presumed secondary to hypertension, presented to the emergency room with one-week history of weakness, fatigue and poor oral intake. The patient was hypotensive (systolic blood pressure of 60 mmHg) and had severe azotemia (serum creatinine of 4.9 mg/dl form baseline of 1.7 mg/dl). Septic shock, methicillin-sensitive *Staphylococcus aureus* bacteremia and oliguric acute kidney injury were diagnosed. The patient was adequately resuscitated with intravenous fluids, vasopressors and broad-spectrum antibiotics, later narrowed to cefazolin. The source of infection was unclear after broad investigation of body fluids and imaging studies. Despite an initial transient clinical improvement, the patient remained in shock with high vasopressor requirements, became anuric and developed acute mental status changes. As a consequence, the decision to start the patient on CRRT was made. We inserted a 13.5 French diameter temporary dual-lumen Quinton-Mahurkar catheter (*Covidien, Inc.*) into the left IJV under US guidance. Although the right IJ is preferred, the intensive care unit team had already inserted a triple-lumen central venous catheter in this site for hemodynamic monitoring and fluid and medication administration. The initial puncture with an 18-gauge needle into the left IJV using real-time US guidance was successful with non-pulsatile back-flow and venous (dark red) blood appearance. Subsequently, after removal of the ultrasound transducer, the rest of the procedure was performed without any resistance to advancing the guidewire and catheter by the Seldinger technique [5]. Good flow of dark red blood was obtained from both catheter lumens. However, the routine post-cannulation chest X-ray (Figure 1) showed that the tip of the catheter was not crossing the midline to the right side and the tip appeared to be lying over the aortic arch suggesting inadvertent carotid artery cannulation. We then obtained blood gas analysis from the catheter which showed: pH of 7.34, partial pressure of oxygen (PO_2_) of 140 mmHg and partial pressure of carbon dioxide (PCO_2_) of 22 mmHg, also suggestive of arterial cannulation. The vascular surgery team was emergently consulted. A pressure transducer was attached to the catheter and showed venous wave forms. To definitively determine the location of the catheter, a Computed Tomography Angiography (CTA) of the head and neck was obtained. The CTA showed the Quinton-Mahurkar catheter extending into the left IJV and subsequently descending to terminate in an anomalous left upper lobe pulmonary vein, just inferior to its anomalous insertion in the left brachiocephalic (innominate) vein (Figures 2A and B). Interestingly, manipulation of the catheter under fluoroscopy by interventional radiology showed that the left brachiocephalic (innominate) vein failed to cross the midline to empty into the right atrium, confirming the venous anomaly. The catheter was therefore removed and a new similar catheter was placed in the right IJV. CRRT was then initiated and continued uneventfully until the patient’s critical condition improved.

## Conclusion

Over 5 million catheters are placed annually in the United States, most of the time for hemodialysis procedures [6]. In comparison to traditional blind CVC insertion techniques using superficial anatomical landmarks, CVC under US guidance achieves higher success rates, including fewer needle attempts, rapid vein localization and fewer complications [7,8]. However, inadvertent arterial trauma or cannulation under US guidance still occurs. Among the various mechanical complications of CVC, unintended arterial puncture has been reported to occur in up to 8% of cases [1]. Because this complication is often recognized by getting pulsatile, bright red blood before the catheter is introduced into the blood vessel, inadvertent arterial catheterization is much less common, <0.1% [2,4]. In most cases of misplacement, the catheter follows an unpredicted pathway into the vena cava tributaries, a complication observed in 40 cases of a series of 2,580 patients. In three of these patients, the aberrant location resulted from a persistent left superior vena cava [9]. The risk factors associated with CVC complications include obesity, short neck and urgent catheterization [4].In patients with hypotension, low hemoglobin and hypoxemia, the visual signs of pulsatile, bright red blood suggestive of arterial puncture might be missed [2,4,10].

When visual discrimination between arterial and venous blood is unreliable, confirmation should be pursued using all available resources (Table 1). One of these is to attach a pressure transducer to the catheter and discriminate between venous and arterial waveforms [10]. This method is not always effective, particularly in patients with severe hypotension, atrial fibrillation, constrictive pericarditis or severe pulmonary hypertension with tricuspid regurgitation [2]. Another method is the use of blood gas analysis, in which high PO_2_ is suggestive of arterial blood. This could be misleading in certain situations. For example, high blood PO_2_ from a central venous catheter was reported in a patient who had the catheter properly placed in the IJV but a shunt in his right arm from his arteriovenous fistula was supplying arterial blood to the superior vena cava via the axillary and subclavian veins [11]. Similarly to our patient, there is a case of unexpected high blood PO_2_ from the dialysis catheter in a patient with anomalous pulmonary vasculature [12]. Although pressure transduction testing and blood gas analysis yield useful information and are easy to perform and obtain, they are not 100% reliable and have to be interpreted in the proper clinical context. Since the complications of arterial cannulation are significant (e.g., massive stroke, hemorrhagic shock) and its delayed recognition could be devastating, confirmatory imaging studies are necessary. Most malpositioned central venous catheters can be identified with frontal and lateral chest radiographs [13]. If uncertainty about the location of the catheter persists after chest radiography, a CTA –if intravenous iodinated contrast is not contraindicated– should be obtained to confirm malposition [14,15].

**Table 1:** Stepwise approach to differentiate true venous placement from inadvertent arterial cannulation following dialysis catheter insertion

1-	Do not remove the catheter
2-	Attach a pressure transducer to the catheter and discriminate between venous and arterial waveforms*
3-	Perform blood gas analysis (high PO_2_ is suggestive of arterial blood)**
4-	Obtain Chest X-ray (frontal and lateral)
5-	If still in doubt, obtain Computed Tomography Angiography***
6-	Surgical or endovascular intervention if arterial cannulation is confirmed

*not effective if severe hypotension, atrial fibrillation, constrictive pericarditis or severe pulmonary hypertension with tricuspid regurgitation.

**not effective in rare cases of anomalous vascular anatomy.

***if intravenous iodinated contrast not contraindicated.

If arterial trauma with a large caliber catheter occurs, prompt surgical or endovascular intervention is likely the safest approach. The pull and pressure technique (removal of the catheter followed by external compression) is associated with significant risk of hematoma, airway obstruction, stroke and pseudoaneurysm, especially when the site of the arterial trauma cannot be effectively compressed. Endovascular treatment appears to be safe for the management of arterial injuries that are difficult to expose surgically, such as those below or behind the clavicle [16].

Our patient had a partial anomalous pulmonary venous connection (PAPVC). PAPVC is a congenital anomaly present in 0.4 to 0.7% of postmortem examinations. About 90% of all PAPVCs originate from the right lung, 7% from the left lung and 3% from both lungs. The common drainage sites are the superior vena cava, the inferior vena cava, right atrium and brachiocephalic (innominate) vein [12,17,18]. Most of these anomalies are discovered incidentally during routine radiographic evaluation of the lungs done for other reasons. In isolated PAPVC, the patient is usually asymptomatic if anomalous venous return is less than 50% of total pulmonary venous blood. Some patients could develop cardio-respiratory symptoms if there is significant left-to-right shunt, which is associated with other cardiac anomalies (10 to 15% of those with atrial septal defects have PAPVC). The majority of patients with a left PAPVC, as in the case of our patient, have a good long term prognosis [12].

In summary, the placement of a dialysis catheter in a vein draining pulmonary venous blood due to anomalous pulmonary venous connection may lead to apparent arterial cannulation (high PO_2_ in blood gas analysis). Our case highlights the available methods to properly identify the catheter location in a patient with a rare congenital pulmonary vascular malformation and the importance of prompt Computed Tomography Angiography for definitive diagnosis before surgical or invasive interventions.

## Consent

The patient was deceased at the time of preparation of this manuscript. Written informed consent was obtained from his next-of-kin for publication of this Case Report and any accompanying images. A copy of the written consent is available for review by the Editor of this journal.

## Competing interests

Dr. Rodan has received a speaker’s fee from Eli Lilly that was not related to this case report. The remaining authors declare that they have no competing interests.

## Authors’ contributions

Analysis of patient’s clinical course and outcomes: JCC, JAN, JP, and ARR; drafting of the manuscript: JCC and JAN; critical revision of the manuscript for important intellectual content: JCC, JAN, and ARR. Administrative, technical, and material support: JCC and JAN. All authors read and approved the final manuscript.

## Pre-publication history

The pre-publication history for this paper can be accessed here:

http://www.biomedcentral.com/1471-2369/15/127/prepub
